# An epidemiological evaluation of pediatric long bone fractures — a retrospective cohort study of 2716 patients from two Swiss tertiary pediatric hospitals

**DOI:** 10.1186/s12887-014-0314-3

**Published:** 2014-12-20

**Authors:** Alexander Joeris, Nicolas Lutz, Bárbara Wicki, Theddy Slongo, Laurent Audigé

**Affiliations:** Department of Pediatric Surgery, Traumatology and Orthopedics, University Hospital (Inselspital) Bern, Freiburgstrasse 15, 3010 Bern, Switzerland; AO Clinical Investigation and Documentation, Stettbachstrasse 6, 8600 Dübendorf, Switzerland; Centre Hospitalier Universitaire Vaudois (CHUV), Rue du Bugnon 46, 1011 Lausanne, Switzerland; Research and Development Department, Schulthess Clinic, Lengghalde 2, 8008 Zürich, Switzerland

**Keywords:** Pediatric, Long bone fracture, Classification, Epidemiology, AO COIAC

## Abstract

**Background:**

Children and adolescents are at high risk of sustaining fractures during growth. Therefore, epidemiological assessment is crucial for fracture prevention. The AO Comprehensive Injury Automatic Classifier (AO COIAC) was used to evaluate epidemiological data of pediatric long bone fractures in a large cohort.

**Methods:**

Data from children and adolescents with long bone fractures sustained between 2009 and 2011, treated at either of two tertiary pediatric surgery hospitals in Switzerland, were retrospectively collected. Fractures were classified according to the AO Pediatric Comprehensive Classification of Long Bone Fractures (PCCF).

Age, sex, BMI, injury and treatment data were recorded. Children were classified into four age classes and five BMI classes were applied. Seven major accident categories were established. Study parameters were tabulated using standard descriptive statistics. The relationship of categorical variables was tested using the chi-square test. The Children’s BMI was compared to WHO reference data and Swiss population data.

**Results:**

For a total of 2716 patients (60% boys), 2807 accidents with 2840 long bone fractures (59% radius/ulna; 21% humerus; 15% tibia/fibula; 5% femur) were documented. Children’s mean age (SD) was 8.2 (4.0) years (6% infants; 26% preschool children; 40% school children; 28% adolescents). Adolescent boys sustained more fractures than girls (p < 0.001). The leading cause of fractures was falls (27%), followed by accidents occurring during leisure activities (25%), at home (14%), on playgrounds (11%), and traffic (11%) and school accidents (8%). There was boy predominance for all accident types except for playground and at home accidents. The distribution of accident types differed according to age classes (p < 0.001). Twenty-six percent of patients were classed as overweight or obese — higher than data published by the WHO for the corresponding ages — with a higher proportion of overweight and obese boys than in the Swiss population (p < 0.0001).

**Conclusion:**

Overall, differences in the fracture distribution were sex and age related. Overweight and obese patients seemed to be at increased risk of sustaining fractures. Our data give valuable input into future development of prevention strategies. The AO PCCF proved to be useful in epidemiological reporting and analysis of pediatric long bone fractures.

## Background

Children are at a high risk of injury with up to one of every four children sustaining an injury annually [1,2]. Fractures are associated with 10% to 25% of these injuries [3], where the lifetime fracture risk is up to 40% for girls and as high as up to 64% for boys [4-9]. With fractures having a considerable impact on the daily living and activity of affected children, they represent an important topic of public health [10,11].

In 2007, the AO Pediatric Comprehensive Classification of Long Bone Fractures (PCCF) [12] was developed and validated according to a 3-phase concept proposed by Audigé et al. [13]. The initial two validation phases showed that the classification process based on radiographic assessment is reliable and accurate [14,15] and that the PCCF system can be considered clinically relevant by pediatric surgeons. The AO Comprehensive Injury Automatic Classifier (AO COIAC) software [16] was developed for testing in a clinical setting following the 3rd and final validation phase, with the purpose of fully documenting and evaluating pediatric long bone fractures, their causes, classification codes, treatments, occurrence of associated complications, and outcomes.

Different risk factors for sustaining fractures in children have been reported, such as age, sex, season, risk-taking behavior, bone mineral density (BMD), sports, but also violence and race/ethnicity and socioeconomic status [4,11,17-22]. Overweight and obesity seem to have an increasing impact [23-28], possibly due to lower bone mass relative to body size, greater mechanical load by falls or reduced body balance [17,20,29-31], and therefore, became major topics of interest for both treating physicians and public health [32].

As the amount of data is still limited, further epidemiological data are needed to better understand the occurrence of pediatric long bone fractures and for the planning of future prevention strategies. The aim of this retrospective cohort study was to review the demographic data of all recorded pediatric long bone fractures at the University Children’s Hospitals in Bern and Lausanne and to evaluate any differences between these data. This is the first time that a large patient cohort was classified according to the AO PCCF using the AO COIAC software to collect epidemiological data on pediatric long bone fractures.

## Methods

The present study was a retrospective cohort study to survey fractures with open physes in children and adolescents younger than 17 years of age in Switzerland. All fractures were sustained between January 2009 and December 2011. Inclusion criteria were documented pediatric long bone fractures in children (in- and outpatient cases) treated at the University Children’s Hospitals in Bern (Inselspital) and Lausanne. Ethical approval from the regional ethic committees (ethical commission canton of Bern, Bern, Switzerland and ethical commission canton of Vaud, Lausanne, Switzerland) was obtained for both clinics. The Children’s Hospital in Lausanne is a tertiary care university hospital, serving as a primary care center for the city of Lausanne, which has approximately 160′000 inhabitants. The Children’s Hospital in Bern is also a tertiary care university hospital, serving as a primary care center for the city of Bern, with a population of 170′000, and the adjacent cities. Being the only children’s hospitals in the aforementioned cities, a majority of children up to the age of 16 are treated in these hospitals (approx. 80%). Twenty-four-hour in- and outpatient primary emergency service is provided to patients for both cities, but also for the entire cantons of Bern and Vaud and the adjoining cantons.

**Figure 1:**
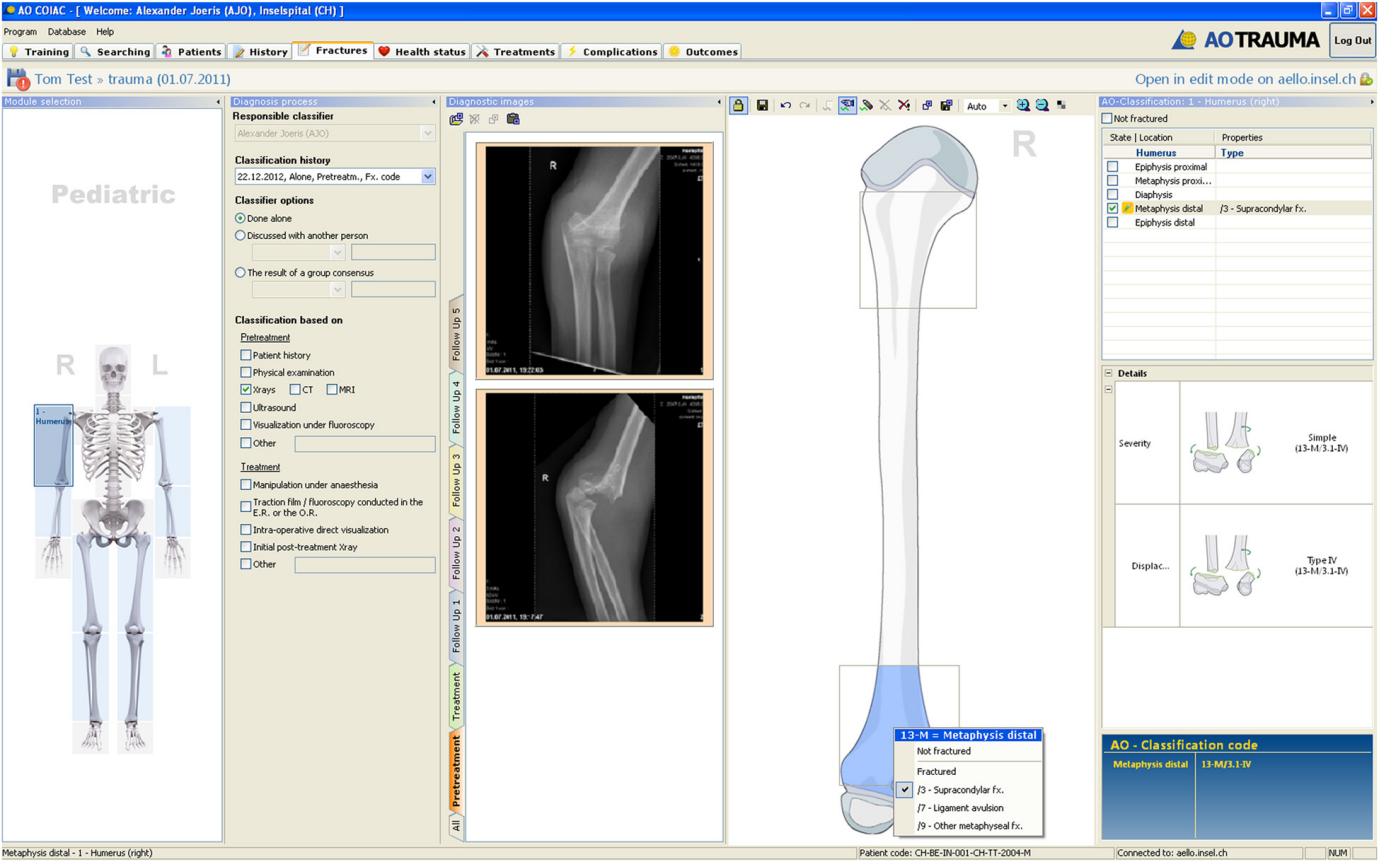
**Screenshot of the AO COIAC interface.** The AO COIAC interface aids through the classification process. To classify a fracture, one can either click on the depicted standard bone or one can draw fracture lines in the bone. Drop down menus and classification options optimize the classification afterwards.

All long bone fractures were classified by an experienced pediatric trauma surgeon in each clinic using the AO PCCF system [15] on the basis of digitalized anteroposterior and lateral view radiographs. Documentation of all classified data was made using the specialized AO COIAC software [33], which facilitates the diagnosis and coding of fractures (Figure 1). In addition to fracture classification, available epidemiological data including age, sex, body mass index (BMI), date and time of injury, cause of injury and data concerning treatment (extracted from digitalized or paper patient charts) were recorded.

Patients were classified into four age groups including: 1) infants (< 2 years); 2) preschool children (2 to < 6 years); 3) school children (6 to < 11 years); and 4) adolescents (11 to < 17 years). The type of accident was divided into seven major categories: 1) home accidents (including all those occurring within the house and yard except on playing devices, e.g. trampoline); 2) school accidents (including accidents occurring at school or kindergarten); 3) playground accidents (including all accidents occurring on public and private playgrounds as well as all accidents with outdoor playing devices); 4) leisure activities; 5) traffic accidents (including all accidents associated with any kind of transportation); 6) falls and 7) others (including long bone fractures due to non-accidental injuries or any undefined accident types). Furthermore, differentiations were made between boys and girls, upper and lower limbs and the time at which fractures were sustained.

For most of the patients undergoing a conventional radiograph at the Children’s Hospital in Bern, height and weight measurements were documented prior to the examination to adapt the individual’s radiation exposure. Therefore, retrospective BMI calculations were possible for these patients. Using this baseline characteristic, the BMI distribution of this subpopulation was compared to the World Health Organization (WHO) BMI-for-age percentiles for boys and girls [34] to further explore BMI as a potential risk factor for pediatric fractures. Severe thinness is defined as a BMI at or above the 3rd percentile and below the 15th percentile for children of the same age and sex; thinness as a BMI at or above the 15th percentile and below the 50th percentile; normal weight children present with a BMI at or above the 50th percentile and below the 85th percentile; overweight children with a BMI at or above the 85th and below the 97th percentile and obesity is defined as a BMI at or above the 97th percentile. For the BMI-for-age percentiles in children < 2 years, the WHO recommends adding or subtracting 0.7 cm from the height before calculating the BMI, depending on whether the child’s height was measured in a standing or lying position, respectively. As it was not known whether the height of children aged < 2 years was measured while standing or lying, BMI calculations were not applicable for this group, and infants were excluded from BMI calculations. In order to compare the proportion of overweight and obese children in our study population to the overall Swiss population in patients aged between 6 and 12 years (as grouped by Swiss surveys before), we exclusively amended our patient group and additionally performed this analysis in children aged 6 to12 years.

All data collected with AO COIAC were transferred into Intercooled Stata version 12 (StataCorp LP, College Station, TX, USA). Study parameters were analyzed and tabulated using standard descriptive statistics. The one-sample test of proportions was used to compare the proportions of overweight and obese children in boys and girls aged between 6 and 12 years in this study with those in boys (18.7%) and girls (17.0%) in the same age category in Switzerland (i.e. with a BMI above the 85^th^ percentile), respectively. The chi-square test was used to assess the relationship between two categorical variables (e.g. BMI classes and age classes).

## Results

### Study collective

**Figure 2:**
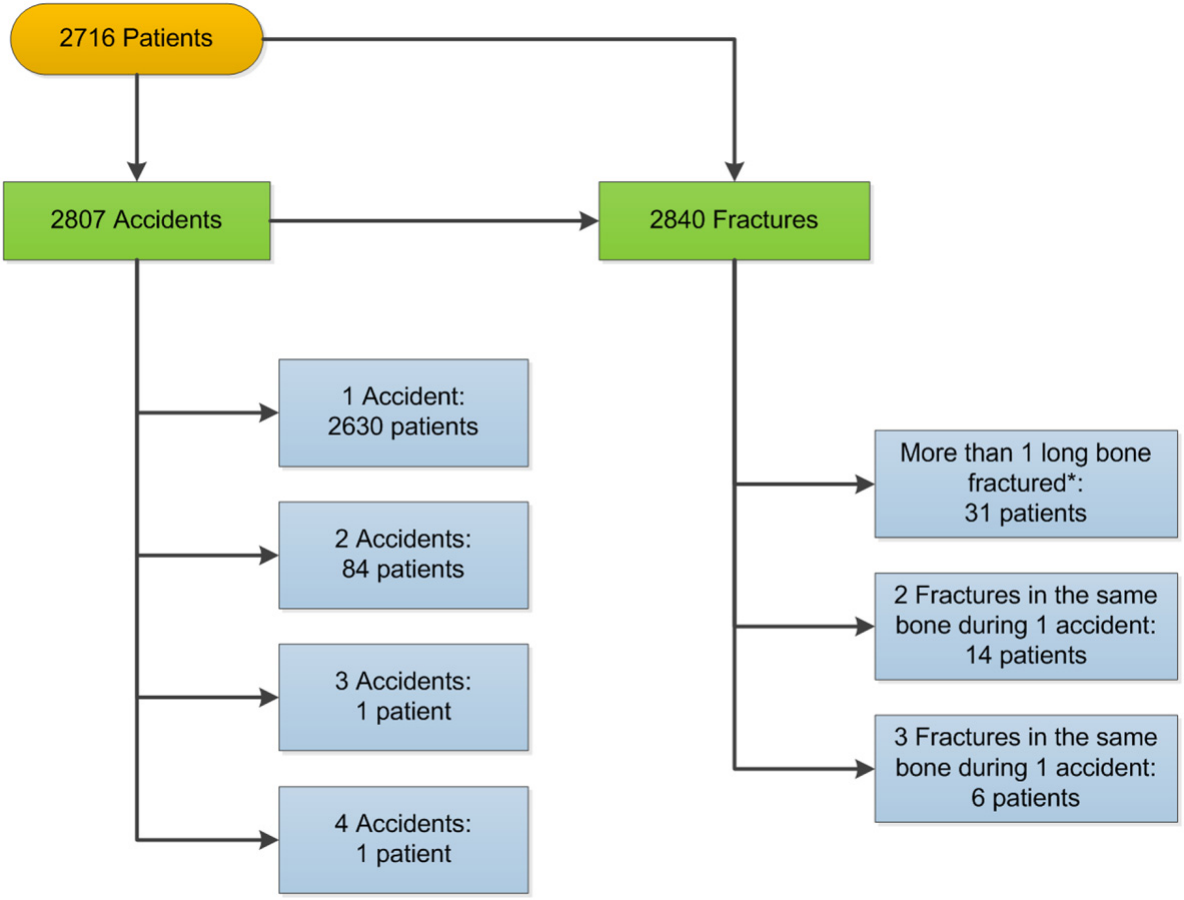
**Overview of patients, accidents and fractures.** Relation between patients, accidents occurred and sustained long bone fractures. * Fractures of the radius and ulna as well as tibia and fibula were considered as one fractured long bone.

A total of 2716 patients (Bern: n = 1066; Lausanne: n = 1650; 60% boys) who experienced 2807 accidents were included into the study. While a single accident was documented for 2630 patients, multiple accidents were recorded for 86 patients. Fifty-one patients sustained more than one fracture during the same accident, either in the same bone (n = 20) or different bones (n = 31) (Figure 2). Of 2840 fractures, 33 (1%) were classified as open fractures according to Gustilo et al. [35,36] and included 23 Grade I fractures, nine Grade II fractures and one Grade III fracture.

According to the predefined age groups, 176 infants (6%), 699 preschool children (26%), 1074 school children (40%) and 767 adolescents (28%) sustained a fracture. The overall mean age was 8.2 years (SD ± 4.0; range 0–17.6 years).

**Figure 3:**
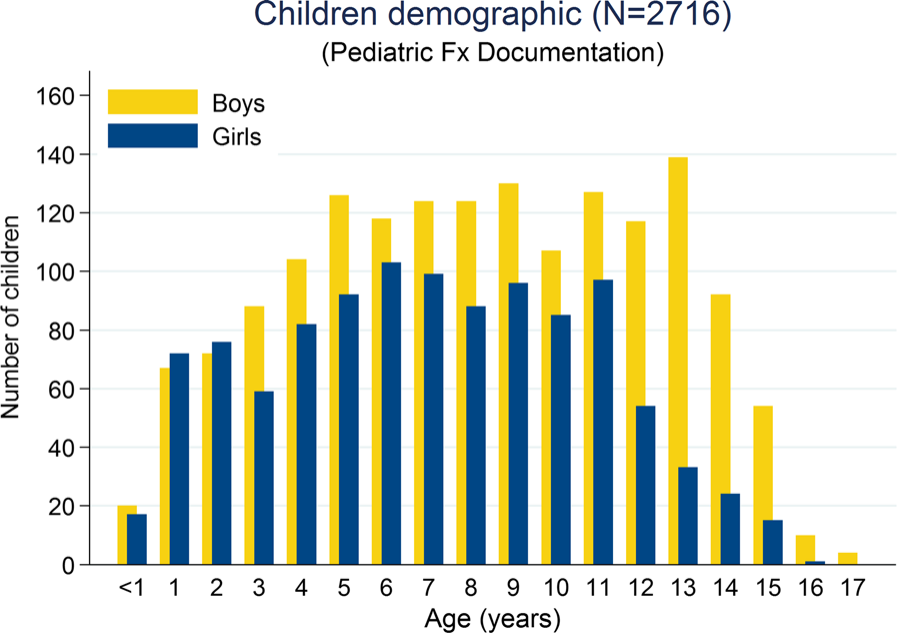
**Age and gender distribution of patients who sustained long bone fractures.** The proportion of boys within age groups increased from 50% in infants to over 70% in adolescents.

More boys sustained a fracture (60%; odds: 1.5:1). In infants, the sex distribution was equal (50% boys, 50% girls), but changed towards a distinct predominance of boys in the adolescent subgroup (71%; odds: 2.4:1; p < 0.001) (Figure 3). Among preschool and school children the proportion of boys was still 56% in each group.

Common concomitant diseases including osteogenesis imperfecta, dysraphism, and neuromuscular or metabolic diseases were reported for 73 patients (2.7%).

### Body mass index

Body mass index data could be analyzed for 791 out of 875 patients from the Children’s Hospital in Bern for whom weight and height measurements were documented. The mean BMI for patients with height and weight measurements was 17.3 kg/m^2^ (SD ± 3.1; range 9.8–34.0). According to the WHO BMI-for-age percentile curves, 210 children (27%) were categorized as either overweight or obese in this study. Whereas the proportion of children with normal weight remained stable (61/62%), the proportion of thin children decreased with age (9% to 3%) while the proportion of overweight children increased with age (12% to 19%; p = 0.018). Accordingly, the highest proportion of overweight patients were adolescents (19%; n = 46) followed by school children (14%; n = 45) and preschool children (12%; n = 30). Obesity was most frequently seen in school children (13%; n = 40) followed by adolescents (10%; n = 24) and preschool children (10%; n = 25) (Table 1). Compared to the overall Swiss 6 to 12 years old (surveyed in 2009), we found a significant higher proportion of overweight and obese boys (33% vs. 18.7%; p < 0.0001) and a non-significant higher proportion of overweight and obese girls (20% vs. 17%; p = 0.12) in this age group. All age groups combined, the proportion of overweight and obese patients was similar whether patients had a fracture in the upper limb (26%) or the lower limb (30%; p = 0.39).

**Table 1:** **Distribution of the pediatric long bone fracture population based on gender and BMI classes in patients from the Children’s Hospital in Bern**

	**Gender**		**WHO BMI classes**					
	**Girls**	**Boys**	**Severe Thinness** ^**1**^	**Thinness** ^**2**^	**Normal** ^**3**^	**Overweight** ^**4**^	**Obesity** ^**5**^	**Total** ^**6**^
Age group	N (%)^7^	N (%)	N (%)	N (%)	N (%)	N (%)	N (%)	N (%)
Infants (< 2 years)	40 (48)	44 (52)	n.a.	n.a.	n.a.	n.a.	n.a.	n.a.
Preschool children (2 to < 6 years)	139 (45)	173 (55)	21 (9)	16 (7)	149 (62)	30 (12)	25 (10)	241 (100)
School children (6 to < 11 years)	165 (44)	214 (56)	9 (3)	27 (9)	192 (61)	45 (14)	40 (13)	313 (100)
Adolescents (11 to 17 years)	95 (33)	196 (67)	7 (3)	15 (6)	145 (61)	46 (19)	24 (10)	237 (100)
Total	439 (41)	627 (59)	37 (5)	58 (7)	486 (62)	121 (15)	89 (11)	791 (100)

WHO = World Health Organization; BMI = Body Mass Index; n.a. = not available.

^1^BMI at or above the 3rd percentile and below the 15th percentile for children of the same sex and age.

^2^BMI at or above the 15th percentile and below the 50th percentile for children of the same sex and age.

^3^BMI at or above the 50th percentile and below the 85th percentile for children of the same sex and age.

^4^BMI at or above the 85th percentile and below the 97th percentile for children of the same sex and age.

^5^BMI at or above the 97th percentile for children of the same sex and age.

^6^Total of patients for whom BMI data could be calculated. For 275 children either height measurements (children under 2 years of age) were incomplete or both height and weight measurements were missing.

^7^Percentage of children within each of the four age groups.

### Type of accident

For 107 (4%) fractures, the injury mechanism was not evaluated due to insufficient documentation in the patient charts. The leading cause for long bone fractures was falls (27%) (Table 2), with a majority of fractures resulting from falling from a height less than 1 meter (57%), followed by falling from an unknown height (22%) and falling from a height greater than 1 meter (21%).

**Table 2:** **Distribution of accident types within sex and age groups**

	**Girls**	**Boys**	**Age (yrs)**	**Infants (< 2 yrs)**	**Pre-school children (2 to < 6 yrs)**	**School children (6 to < 11 yrs)**	**Adolescents (11 to 17 yrs)**	**Total**
Type of accident	N (%)	N (%)	Mean	N (%)	N (%)	N (%)	N (%)	N
School/kindergarten	84 (8)	148 (9)	9.9	4 (2)	25 (3)	103 (9)	100 (13)	232 (8)
Playground	152 (14)	168 (10)	6.6	20 (11)	128 (18)	136 (12)	36 (5)	320 (11)
Leisure activities^1^	227 (20)	468 (28)	10.3	4 (2)	79 (11)	291 (26)	321 (40)	695 (25)
Fall	312 (28)	449 (27)	7.7	38 (22)	220 (30)	353 (32)	150 (19)	761 (27)
Traffic	121 (11)	191 (11)	9.3	4 (2)	71 (10)	118 (11)	119 (15)	312 (11)
At home	174 (16)	206 (12)	4.9	92 (52)	179 (25)	71 (6)	38 (5)	380 (14)
Other^2^	45 (4)	62 (4)	8.0	14 (8)	20 (3)	41 (4)	32 (4)	107 (4)
Total	1115 (100)	1692 (100)	8.2	176 (100)	722 (100)	1113 (100)	796 (100)	2807 (100)

^1^ Including a total of 153 sports club activities.

^2^ Not evaluated due to insufficient documentation in the patient charts.

Leisure activities were the second most frequent cause of fractures (25%; n = 695), with those activities pursued in organized sports clubs, representing 5% (n = 153) of all accidents. Out of the 153 accidents sustained in sports clubs, 90% (n = 138) involved boys and almost three-quarters (73%; n = 112) occurred while playing soccer. Overall, playing soccer was the main reason for sustaining a long bone fracture during leisure activities (26%; n = 182) (Table 3).

**Table 3:** **Specific activities associated with the occurrence of pediatric fractures during leisure activities**

**Type of leisure activity accident**	**Infants (< 2 yrs)**	**Pre-school children (2 to < 6 yrs)**	**School children (6 to < 11 yrs)**	**Adolescents (11 to 17 yrs)**	**Total**
	**N**	**N (%)**	**N (%)**	**N (%)**	**N (%)**
Soccer^1^	-	5 (6)	72 (25)	105 (33)	182 (26)
Skiing	2	35 (44)	40 (14)	38 (12)	115 (17)
Rollerblade	-	3 (4)	35 (12)	23 (7)	61 (9)
Ice skating/ice hockey	-	7 (9)	25 (9)	27 (8)	59 (8)
Unclassified leisure activities	1	7 (9)	23 (8)	19 (6)	50 (7)
Horse	-	5 (6)	22 (8)	11 (3)	38 (5)
Ball against hand	-	-	21 (7)	13 (4)	34 (5)
Snowboard	-	-	5 (2)	27 (8)	32 (5)
Skateboard	-	2	10 (3)	14 (4)	26 (4)
Sledding	-	5 (6)	12 (4)	9 (3)	26 (4)
Running	1	6 (8)	7 (2)	5 (2)	19 (3)
Gymnastic	-	3	4 (1)	7 (2)	14 (2)
Judo	-	-	5 (2)	7 (2)	12 (2)
Basketball	-	-	3	3	6 (1)
Motocross	-	-	2	3	5 (1)
Rugby	-	-	1	4	5 (1)
Mountain bike	-	-	-	2	2
Uni hockey	-	-	-	2	2
Badminton	-	-	1	-	1
Handball	-	-	1	-	1
Rings	-	-	-	1	1
Schwingen^2^	-	-	1	-	1
Tennis	-	-	1	-	1
Unicycle	-	-	-	1	1
Table tennis	-	1	-	-	1
Total	4	79 (100)	291 (100)	323 (100)	695 (100)

^1^ Soccer accidents including 111 soccer related fractures during club-sport-activities.

^2^ Style of folk wrestling native to Switzerland.

Accidents occurring at home were the third most frequent cause of fractures (14%), followed by playground accidents (11%), traffic accidents (11%) and school accidents (8%). Playground accidents occurred mainly whilst playing on swings (32%), slides (29%) and trampolines (22%) (Table 4). Bicycle (45%) and non-motorized scooter accidents (35%) represented the main modes for sustaining traffic accident-related fractures; on the other hand, 11% of the pediatric population sustained a fracture as a pedestrian with only 3% injured as car passengers (Table 5). Nearly two thirds of all fractures sustained at school occurred whilst undertaking school sport activities (64%). Playground accidents and accidents at home occurred equally for boys and girls, while the remaining accident types (school, leisure activities, fall, traffic, at home and others) reflected the overall sex distribution of 60% boys and 40% girls (Table 2).

**Table 4:** **Specific activities associated with the occurrence of pediatric fractures on playgrounds**

**Type of playground accident**	**Infants (<2 yrs)**	**Pre-school children (2 to < 6 yrs)**	**School children (6 to < 11 yrs)**	**Adolescents (11 to 17 yrs)**	**Total**
	**N (%)**	**N (%)**	**N (%)**	**N (%)**	**N (%)**
Swing	5 (25)	34 (27)	52 (38)	11 (31)	102 (32)
Slide	13 (65)	52 (41)	24 (18)	3 (8)	92 (29)
Trampoline	-	25 (20)	35 (26)	9 (25)	69 (22)
Climbing	-	11 (9)	16 (12)	5 (14)	32 (10)
Turntable	1	2	7 (5)	2	12 (4)
Carousel	1	1	2	5 (14)	9 (3)
Seesaw	-	1	-	1	2
Playing device	-	1	-	-	1
Wall bars	-	1	-	-	1
Total	20 (100)	128 (100)	136 (100)	36 (100)	320 (100)

**Table 5:** **Specific activities associated with the occurrence of pediatric fractures in traffic**

**Type of traffic accident**	**Infants (< 2 yrs)**	**Pre-school children (2 to < 6 yrs)**	**School children (6 to < 11 yrs)**	**Adolescents (11 to 17 yrs)**	**Total**
	**N (%)**	**N (%)**	**N (%)**	**N (%)**	**N (%)**
Bicycle	1	40 (56)	41 (35)	58 (49)	140 (45)
Non-motorized scooter	1	15 (21)	56 (47)	38 (32)	110 (35)
Pedestrian	1	7 (10)	19 (16)	10 (8)	37 (12)
Car passenger	-	7 (10)	2	1	10 (3)
Motor scooter driver	1	-	-	9 (8)	10 (3)
Motor scooter passenger	-	1	-	2	3
Other	-	1	-	1	2
Total	4	71 (100)	118 (100)	119 (100)	312 (100)

**Figure 4:**
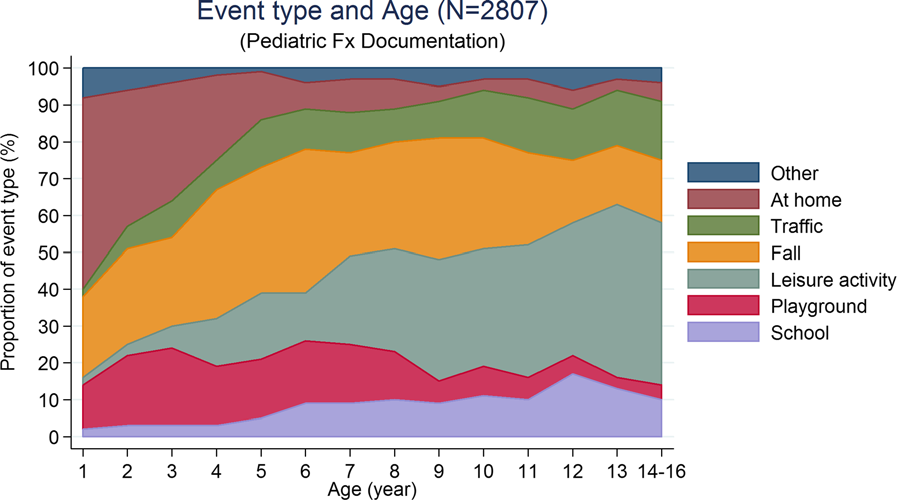
**Accident types correlated to age.** There is a predominance of fractures due to accidents at home throughout the first four years of life, whereas during school-age and adolescence leisure activities become the leading cause.

Fractures sustained from school, leisure activities and traffic accidents were associated with higher proportions of patients with increasing age, whereas home accident-related long bone fractures, being the leading cause of fractures in infants (52%) and preschool children (25%), were significantly less frequent in school children and adolescents (6% and 5%, respectively) (p < 0.001). Fractures sustained from a fall increased in preschool and school children, but became less frequent in adolescents (Figure 4).

A high proportion of fractures caused by undertaking leisure-activities (33%) was observed during the winter months (i.e. December to March); 232 fractures were sustained from skiing, snowboarding, ice skating or sledding, with 50% caused by skiing accidents. For all skiing accident-related fractures, there was an approximately equal distribution among preschool children (n = 35), school children (n = 40) and adolescents (n = 38). Fractures caused by playground accidents occurred mainly between March and October, while no seasonal distribution was observed for school accidents.

### Fracture distribution

The most commonly reported long bone fractures involved those of the forearm (59%; n = 1690) followed by fractures of the upper arm (21%; n = 602), the lower leg (15%; n = 413) and the femur (5%; n = 135) (Table 6). Forearm fractures were significantly more frequent in school children (65%) and adolescents (63%) compared to infants and preschool children (42% and 50%, respectively). Femoral fractures were more frequently observed in the infant group (13%) compared to around 3% to 6% for the other age groups. Femoral fractures were mostly reported for boys (72%), whereas all other long bone fracture types were distributed equally between boys and girls (p = 0.011). Fractures of the radius and ulna represented the largest proportion (around 60%) within each accident category, except for home accidents (53%). Playground and leisure activity categories included several activities (e.g. climbing on playgrounds, soccer, snowboarding, ice skating or running) where forearm fractures accounted for more than 70% of all fractures sustained. Furthermore, within the traffic category, 70% of all fractures involving non-motorized scooters were fractures of the radius and ulna.

**Table 6:** **Long bone fracture distribution within age groups and sex**

	**Infants (<2 yrs)**		**Pre-school children (2 to < 6 yrs)**		**School children (6 to < 11 yrs)**		**Adolescents (11 to 17 yrs)**		**Total**
	**Girls**	**Boys**	**Girls**	**Boys**	**Girls**	**Boys**	**Girls**	**Boys**	
Bone	N (%)	N (%)	N (%)	N (%)	N (%)	N (%)	N (%)	N (%)	N (%)
Humerus	14 (16)	11 (13)	97 (31)	130 (32)	112 (23)	131 (21)	35 (15)	72 (13)	602 (21)
Radius/ulna	39 (43)	35 (40)	172 (55)	192 (46)	310 (64)	425 (67)	142 (60)	375 (65)	1690 (59)
Femur	7 (8)	16 (18)	10 (3)	30 (7)	17 (3)	18 (3)	4 (2)	33 (6)	135 (5)
Tibia/fibula	30 (33)	25 (29)	36 (11)	60 (15)	49 (10)	63 (10)	54 (23)	96 (17)	413 (15)
Total	90 (100)	87 (100)	315 (100)	412 (100)	488 (100)	637 (100)	235 (100)	576 (100)	2840 (100)

Of all lower leg fractures occurring during leisure activities, 51% resulted from skiing accidents. The distribution of fractured bones significantly differed among the types of accidents (p < 0.001).

Only 3 long bone fractures were documented as related to non-accidental injuries.

## Discussion

In the presented study we were able to describe distinct age and sex related differences in the fractured bone distribution and different accident types. Furthermore, compared with the overall Swiss population of similar age, we identified a higher percentage of overweight and obese children in our study population. This observation suggests that overweight and obese children are at increased risk of sustaining a fracture.

While the overall predominance of fractures sustained by boys over girls with odds of 1.5:1 was consistent with current knowledge [11,20], these odds varied according to our predefined age groups. Biological and behavioral differences related to sex and age are thought to particularly explain the male predominance in the adolescent patient group. BMD decreases towards the age of pubertal peak height velocity with girls experiencing this peak earlier than boys [37]. This biological effect might contribute to the male predominance in adolescents in this study and reflects the finding of Faulkner et al. that the decrease in BMD in adolescents did coincide with an increase of accidents while accidents decreased as BMD rebounded after peak height velocity [37]. Furthermore, a behavioral change in the types of general activities undertaken as well as the level of risk taking can be observed with increasing age.

In accordance with published data, boys were more likely to sustain femoral fractures [38,39]. However, we did not observe a bimodal distribution of these fracture types with peaks around the ages of 2 to 5 and 14 to 17 years as reported by Hinton et al. [40]. One explanation for the lack of the second peak could be the small amount of motor vehicle accident-related long bone fractures reported in this study compared to other studies [39-41]. High-energy injuries, especially those sustained during motor vehicle accidents, are thought to be the main reason for femoral fractures in adolescents [39,41].

Petersen et al. reported a doubling in the prevalence of overweight and obese children and adolescents from 1986 to 2001, reaching 23.1% (18.3% overweight and 4.8% obese children) at or above the 85^th^ BMI-for-age related percentile [42]. Our study population showed a slightly higher proportion that significantly increased with increasing age; this observation may reflect the known global trend of mounting numbers of overweight and obese children and adolescents as predicted by the WHO [32], but more likely reflects the relationship between overweight status and fracture risk.

National Swiss surveys found a decrease in the number of overweight and obese children between 2002 and 2007 following a stabilization in the prevalence of childhood obesity in 2009; the prevalence of overweight and obesity in children from 6 to 12 years was 19.9% for boys and 18.9% for girls in 2002 compared to 18.7% for boys and 17.0% for girls in 2009 [43,44]. Compared to the overall Swiss population, the proportions of overweight and obese girls and boys in the same age group were higher in this study, with a significantly higher proportion of overweight boys (33%; p < 0.0001) and a clear tendency but not significantly higher proportion in girls (20%; p = 0.12). These findings may additionally point towards an increased risk for overweight and obese children for sustaining a fracture.

Kessler et al. [45] reported a statistically significantly higher proportion of lower limb fractures in overweight and obese children. Although we made a similar observation, this relation was not significant in our study.

Though categorized differently, our data confirmed recently published data reporting falls being the leading cause for fractures in children and adolescents (27% in this study) with most of the fractures resulting from a fall from low height (57% within this group) [22].

Children older than 5 years sustain more sport-related injuries as they start to pursue more organized sports activities (i.e. in sports clubs) around this age [20]. There was a shift from home- and playground-related fractures occurring in infants and preschool children towards fractures resulting from sports activities undertaken by the older age groups of our study population. Due to the retrospective data collection, we could not reliably determine whether the respective sports activities were organized or not. The manner in which the “accident type” was categorized is not recognized as standard, although attempts were made to match previous reports to facilitate the comparison of results. While this may be imperfect, sports club-related long bone fractures defined in our population were reported more often for school age and adolescent patients and reflect the above mentioned.

During the winter months a seasonal peak (33%) of winter leisure activity-related fractures was observed at both Children’s Hospitals, Bern and Lausanne.

Consistent with previous reports [46,47], forearm fractures were mainly associated with snowboard accidents, whereas lower leg fractures were mostly sustained while skiing. Moreover, 49% of all lower leg fractures in the ‘leisure activities’ category were sustained while skiing, and skiing was the main reason for lower leg fractures. The risk of sustaining a fracture during snowboarding has been reported to be twice the risk of sustaining a fracture during skiing [17,48]. In our study population, there were 3.5 times more fractures following skiing than following snowboarding; however, this might be explained by the 4.8 times higher proportion of the Swiss population practicing skiing compared to snowboarding [49].

The rising popularity of trampolines in yards and playgrounds has been followed by an increase in trampoline-related injuries impacting the physical health of children and adolescents [50]. Our study supported this assumption, as together with accidents on slides and swings, trampoline accidents represented one of the three leading causes for playground activity related fractures.

The increased popularity of non-motorized scooters since the beginning of this century [51], especially for school age children and adolescents, was reflected in our study. Besides cycling, non-motorized scooters were the second most frequently reported cause for long bone fractures sustained during traffic accidents.

Finally, we have to acknowledge that our rate of non-accidental injuries is rather low with only three patients. Most likely, this is due to underreporting and our study focusing on long-bone fractures, but not on non-accidental injuries in general.

This study has its limitations, in particular, its retrospective study design. Data quality was dependent on the completeness of the patient charts, specifically regarding the accident type. The categorization of pediatric accident data is often prone to error or misclassification because younger children, in particular, may have difficulties in explaining the correct course of events or the accident occurred unobserved. The fact that approximately 80% of all pediatric long bone fractures are treated in the Children’s hospitals in Bern and Lausanne could lead to potential publication biaz assuming that the remaining 20% of long bone fractures could present differently. As the two hospitals do not cover 100% of patients of the respective regions, epidemiological data such as incidences could not be provided. Nonetheless, the large cohort is a strength of this study.

## Conclusion

In conclusion, our retrospective cohort study provided valuable epidemiological data about pediatric long bone fractures in a large Swiss cohort of children and adolescents and may contribute to the development of appropriate prevention strategies in the future. Comprehensive epidemiological data could also serve as a future teaching aid to orthopedic and trauma surgeons in training.

The AO PCCF system was successfully used to classify all encountered fractures; the detailed fracture patterns however were beyond the scope of this paper. The AO COIAC software proved to be practical and useful in the clinical context, providing the basis for a standardized, multicenter documentation of pediatric long bone fractures. We encourage any hospital treating pediatric fractures to adopt the AO PCCF system and use the freely available AO COIAC software [16].
